# Anatomy of Mammalian Replication Domains

**DOI:** 10.3390/genes8040110

**Published:** 2017-03-28

**Authors:** Shin-ichiro Takebayashi, Masato Ogata, Katsuzumi Okumura

**Affiliations:** 1 Department of Biochemistry and Proteomics, Graduate School of Medicine, Mie University, Tsu, Mie 514-8507, Japan; ogata@doc.medic.mie-u.ac.jp; 2 Laboratory of Molecular and Cellular Biology, Department of Life Sciences, Graduate School of Bioresources, Mie University, Tsu, Mie 514-8507, Japan

**Keywords:** DNA replication, mammalian chromosome, replication domain, replication foci, replication origin

## Abstract

Genetic information is faithfully copied by DNA replication through many rounds of cell division. In mammals, DNA is replicated in Mb-sized chromosomal units called “replication domains.” While genome-wide maps in multiple cell types and disease states have uncovered both dynamic and static properties of replication domains, we are still in the process of understanding the mechanisms that give rise to these properties. A better understanding of the molecular basis of replication domain regulation will bring new insights into chromosome structure and function.

## 1. Introduction

**Figure 1:**
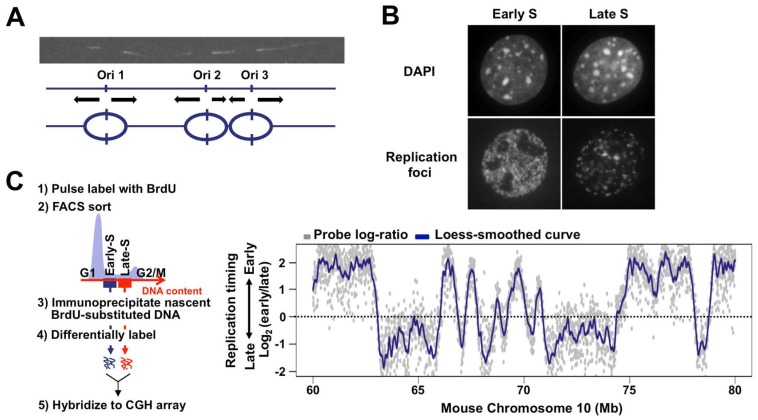
DNA replication in mammalian cells analyzed by different methodologies. (**A**) Multi-replicon structure of mammalian cells revealed by the DNA fiber technique. The replicating cellular DNA was labeled with biotin-dUTP by the bead-loading method and detected with avidin-FITC on DNA fibers extended from the cell nucleus [17]. Three origins (indicated by the vertical arrows) were presumed to be activated simultaneously. To label replicating DNA, nucleoside analogues such as BrdU can also be used [3]; (**B**) Patterns of replication foci observed in early and late S phase of mammalian cells. Sites of DNA synthesis in the nucleus were visualized by the incorporation of biotin-dUTP and subsequent detection with avidin-FITC (top) [18]. Cellular DNA was stained with DAPI (bottom); (**C**) Flow chart of genome-wide replication domain analysis. Unsynchronized cells are pulse-labeled with BrdU. BrdU-substituted DNA from early and late S phase fractions are collected, differentially labeled, and hybridized to a whole-genome CGH array [19]. Alternatively, BrdU-substituted DNA from each fraction can be subjected to NGS (**left**) [20]. Exemplary replication domain organization from mouse embryonic stem cells for a 20 Mb region of chromosome 10 [21]. Log_2_(early/late) raw values (the signal ratio of early and late replicating DNA as shown in grey dots) for each CGH probe are plotted against the chromosomal position. Loess-smoothed plot is shown in blue.

In mammals, many potential replication origins are distributed throughout the genome. Replication forks from selectively activated origins proceed at approximately 1–2 Kb/min, which enables mammalian genomes to be replicated in an 8–10 h S phase. If each mammalian chromosome consisted of only a single replication origin like the bacterial genome, it would take nearly a month to complete duplication of the entire chromosome. Early pioneering work that directly visualized DNA replication on DNA fibers revealed the multi-replicon structure of the mammalian genome [1,2]. Several adjacent origins spaced up to several hundred Kb are activated in a relatively synchronous manner, suggesting that DNA replication takes place in large chromosomal units [3] (Figure 1A). Temporal order of DNA replication in S phase is first established at the level of these large chromosomal units during early G1 phase, and subsequent selection of origins to be fired occurs within each chromosomal unit [4,5,6], suggesting the functional significance of this structural unit in the control of mammalian DNA replication. However, it has long been difficult to gain further insight into this structural unit of mammalian DNA replication due to a lack of methodologies that allow analysis at the molecular level. In the nucleus, replication sites can be visualized by the incorporation of nucleoside and nucleotide analogues into replicating DNA as a discrete structure called “replication foci,” whose relationship with the replication unit revealed by DNA fiber experiments is not fully understood [1]. Intranuclear distribution patterns of replication foci change dynamically during S phase: chromosomal regions in the interior of the nucleus are replicated in early S phase, while regions at the nuclear periphery are replicated in late S phase [7,8,9] (Figure 1B). Spatio-temporal regulation of replication sites has been of great interest in association with chromosome structure and function, though this type of cytological approach did not provide an answer as to which chromosomal segments are replicated in early and late S phase. However, recent technological and methodological advances have enabled genome-wide mapping of structural units of chromosomal DNA replication, now called “replication domains” and opened new avenues for DNA replication research [10,11,12,13]. Intriguingly, early and late replication domains are largely consistent with A and B compartments of self-interacting chromatin domains revealed by the chromosome conformation capture method [14,15,16], suggesting that replication domains represent a fundamental unit of mammalian chromosome structure. In this review, we discuss what is known and not known about the structural properties of mammalian replication domains based on newly obtained genome-wide data as well as the previous cytological data.

## 2. The Mammalian Replication Domain Comes into Focus

**Figure 2:**
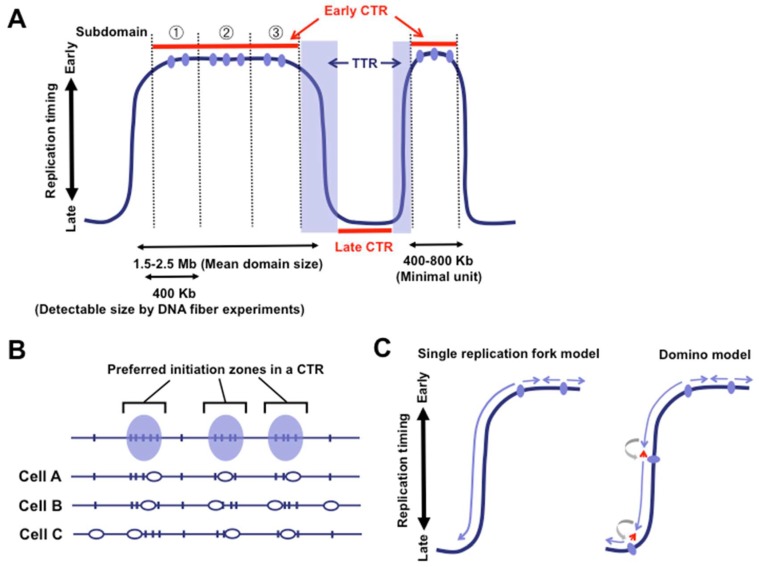
Schematic diagram of mammalian replication domain structures. (**A**) A chromosome consists of early and late CTRs delimited by TTRs. The mean size of regulated replication domains is 400–800 Kb, suggesting that several adjacent sub-domains may form a larger Mb-sized domain. Visualizing the whole replication process (including origin firing and fork progression) of a Mb-sized domain by DNA fiber techniques is technically challenging since the average fiber length that can be prepared is generally limited to 400 Kb; (**B**) Many potential origins exist within a CTR and a different set of origins is fired in each cell. Some sets of origins are found in groups to form preferred initiation zones (highlighted as blue ovals); (**C**) Two possible models for replication regulation at TTRs. A single unidirectional fork from the origin at the edge of the early CTR travels across several hundred Kb toward the late CTR without any new origin firing (**left**). Fork progression from the early CTR triggers the sequential activation of subsequent origins in TTRs in a domino effect (**right**).

Over the last decade, several methods have been developed to map replication domain structure at the genome-wide level in various human and mouse cell types. David Gilbert and colleagues devised a method in which BrdU-labeled replicating DNA is immunoprecipiated from FACS-sorted early and late S phase cells and the quantitative ratio between them (early vs. late) at each chromosomal segment is determined genome-wide using microarrays or next-generation sequencing (NGS) technologies [19,20] (Figure 1C). The resulting replication profiles revealed that chromosomes are mosaic structures of Mb-sized early and late replicating domains (1.5–2.5 Mb mean size) separated by relatively sharp boundaries [12] (Figure 1C). The regions with similar replication timing and boundaries between them are designated as “constant timing regions (CTRs)” and “timing transition regions (TTRs),” respectively (Figure 2A). These structural features of replication domains are independent of the methodology used, since almost indistinguishable replication domain structures have also been reported by detecting copy number differences arising before and after DNA replication [10]. Thus, new genome-wide methodologies enabled sequence level identification of early and late replication units that have only been cytogenetically approachable for several decades and uncovered both static and dynamic structural properties of replication domains.

## 3. Flexible Nature of Replication Origins in CTRs

In mammals, many potential origins are distributed throughout the genome. Genome-wide short nascent strand mapping in embryonic stem (ES) cell populations revealed that origin density is 25–40 origins/Mb [22]. However, single molecule analysis to directly visualize replication of DNA fibers revealed that only a subset of potential origins is actually used in a given S phase. Individual cells in the same population use different sets of origins, and more surprisingly, the same cell uses different sets of origins from one cell cycle to the next [17,23,24]. The average distance between two adjacent active origins estimated by DNA fiber experiments is ~150 Kb, though this might be underestimated by technical limitations. While the analysis of origin activation at the single molecule level is feasible, the detected origin to origin (ori-to-ori) distance is known to be largely dependent on the fiber length [25]. Taking into consideration the fact that the average fiber length is generally ~400 Kb [24,26], ori-to-ori distances larger than that are often obscured in this experimental condition. Similarly, estimates of the number of activated origins that form a CTR might also be affected by fiber length. In addition to DNA fiber length bias, labeling periods might also significantly affect the measurement. For instance, longer labeling periods would fail to detect replication fork movement whose activation and termination occurs within a short period. These technical limitations make it difficult to know the exact percentage of origins actually used in a given S phase. Nonetheless, the fact remains that origin usage is variable in individual cells.

Despite stochastic activation, origins are often grouped in specific regions, contributing to preferred initiation zones within individual CTRs (Figure 2B). The positions of potential replication origins are highly conserved among different cell lines, but each cell line seems to use these origins with different frequencies [27]. Origins are not uniformly distributed with respect to replication timing. It has been shown that origin density is significantly lower in late domains compared with early domains [27], which may be reflected as relatively unstructured and more stochastic replication in late domains [28]. However, low origin density does not necessarily mean that late replication domains need more time to be duplicated, since the rate of replication fork movement is faster in late replication domains (1.5–2.3 Kb/min) than that of early replication domains (1.1–1.2 Kb/min) [18]. The biological significance of this flexible origin firing within CTRs remains elusive, though this brings about a situation in which a gene-coding strand is replicated as the leading strand in one cell while the same strand is replicated as the lagging strand in another cell. It has been shown that replication fork progression is significantly co-oriented with transcription in mammalian cells [29]. In bacteria, the effect on transcription is different between head-on collision (i.e., replication and transcription machineries move in opposite directions) and co-directional collision [30], while in mammals, the existence and extent of such interactions between replication and transcription machineries are not well understood.

Several factors such as chromatin structure and specific DNA sequences that form G-quadruplexes are thought to regulate origin firing [6,31,32]. In yeast, long-range chromatin interaction mediated by transcription factors Fkh1 and Fkh2 controls timing of origin firing [33]. In mammalian cells, selection of origins used in each S phase occurs at a discrete time point during G1 phase called the origin decision point (ODP) [4,6]. Replication timing of microscopically observable large chromosomal units is re-established in each cell cycle at another time point during G1 phase called the timing decision point (TDP) [5,6]. These two processes are temporally separable. Intriguingly, the TDP precedes the ODP, indicating that the replication timing program of large chromosomal units (possibly replication domains) is established prior to origin selection. Although this does not necessarily mean that the regulation of individual origin firing timing is mechanistically uncoupled from domain-wide replication timing regulation, there are indeed some cases where local changes in origin firing program are not sufficient to induce a domain-wide switch in replication timing. For instance, forced tethering of histone acetyltransferases (HATs) and histone deacetylases (HDACs) to the human beta-globin origin results in advanced and delayed firing of the inserted origin, respectively, but observed changes in replication timing is only partial (~20% of total S phase length) [34].

## 4. TTRs: One-Way Roads?

What about origins in TTRs delimiting early and late replication domains? Recent genome-wide origin mapping shows a sharp decline in the origin density from early to middle/late replicating regions [27], suggesting that TTRs are origin-poor regions. When examining replication domain data, one can easily imagine that there is something different about origin regulation at TTRs. In contrast to CTRs, TTRs have clear unidirectionality in replication progression from early to late domains over several hundred Kb without any bump in the profile. Unidirectional nature of replication progression at TTRs is further supported by a recent study that performed genome-wide mapping of highly purified Okazaki fragments [29]. While many forks in a replication domain seem to terminate their replication by meeting with forks from neighboring origins during the first 1–2 h of S phase, forks from the edge of the domain might continue to grow for several hours. This view is supported by the DNA fiber experiment showing that very few origin firing events occur in a TTR formed in the mouse large *Igh* locus (~3 Mb) of non-B cells [35]. However, in pro-and pre-B cell lines, the entire locus is replicated during early S phase and firing of multiple origins is observed throughout the locus, suggesting that suppression of origin firing leads to the formation of a TTR in non-B cells. Furthermore, insertion of ectopic origins into the TTR of the *Igh* locus resulted in poor firing efficiency. Currently the mechanism behind this phenomenon remains largely unclear, except that the insertion of an active transcription unit that brings about several euchromatic histone modifications is not sufficient to induce origin firing in the *Igh* TTR. The extent to which findings from the *Igh* TTR can be applied to others is also unclear. These observations, however, do not necessarily require that a single replication fork moves unidirectionally across several hundred Kb from early to late domains (Figure 2C left). Alternatively, sequential activation of a few origins could occur in a domino-like fashion from the early to the late side of the TTRs [36] (Figure 2C right). In this case, the unidirectional fork from an early domain triggers activation of the downstream origin. Forks from the activated origins progress bidirectionally, though one of them terminates its progression soon by meeting with the fork from an earlier activated neighboring origin (red arrows in Figure 2C right), which produces very short labeling tracks in the DNA fiber experiments. Such short labeling tracks may often merge with longer tracks derived from neighboring forks during the period of labeling, thus making them difficult to be detected. The fork on the other side keeps extending until it triggers activation of another origin further downstream in the same fashion. This domino-like sequential activation of origins would also create the TTRs seen in the genome-wide profiles. Unidirectional forks that travel for several hours from early to late domains would increase the chance of fork stalling and collapse, while domino-like sequential activation of origins would overcome such problematic situations. In either model, the size of chromosomal segment that (almost) unidirectional forks can replicate during S phase is limited, explaining the formation of relatively sharp boundaries at the TTRs.

## 5. Dynamic Properties of Replication Domains

It has been shown that up to 20% of the genome undergoes replication domain reorganization during ES cell differentiation into neural progenitor cells [12]. Further comprehensive analyses in various mouse cell types revealed that at least 50% of the genome undergoes replication domain reorganization during development [37]. This raised the possibility that replication domain organization is highly cell-type specific. Indeed, closely related mouse ES cells and epiblast stem cells are distinguishable based on differences in replication domain organization [15,37,38]. While Mb-sized replication domains are frequently detected, the size of replication domain switching from either early-to-late (EtoL) and late-to-early (LtoE) usually falls into a 400–800 Kb range, which is well conserved between human and mouse. The relatively small size of developmentally regulated domains may explain why conventional replication (BrdU)-banding on metaphase chromosomes has failed to detect cell-type specific replication profiles. Given that the regulated domain size is 400–800 Kb, domains much larger than this size may consist of multiple sub-replication domains (Figure 2A). Generally, gene density and transcriptional activity are higher in early CTRs compared with TTRs and late CTRs, though there is not a simple correlation between gene expression changes and replication domain reorganization during cell differentiation [39,40,41].

Currently it is largely unknown what is regulating these “developmental domains.” Intriguingly, developmental domains regardless of their replication timing share some structural properties with late replication domains. For instance, MNase-sensitivity of early replicating domains is generally high compared with late domains, but EtoL and LtoE domains possess MNase-insensitive chromatin reminiscent of late domains even when they are early replicating [42]. The same is true for replication origin density in developmental domains [27]. Hence, the forces driving developmental domains to behave like early domains while keeping some of the late domain properties seem to be involved in the regulation of developmental domains. Deficiency in the chromatin remodeling esBAF complex subunits has shown to induce late replication in a very small subset of ES cell-specific early replication domains [21]. Since the majority of ES cell-specific early replication domains are not affected by the loss of esBAF subunits, the mechanism that maintains early replication of EtoL developmental domains may vary from domain to domain. Epigenetic mechanisms might be involved in developmental regulation of replication domains, though mutation of several epigenetic modifiers exhibit little or no effect on the organization of replication domains including developmental ones [16,43]. Considering that aberrant expression of a number of genes is induced by these epigenetic modifier mutations, gene transcription might not be sufficient to drive replication domain reorganization. Thus, our understanding on developmental domains is still preliminary and further studies are necessary.

## 6. Replication Domains, Replication Foci, and Replication Origins: Making All Things Consistent

**Figure 3:**
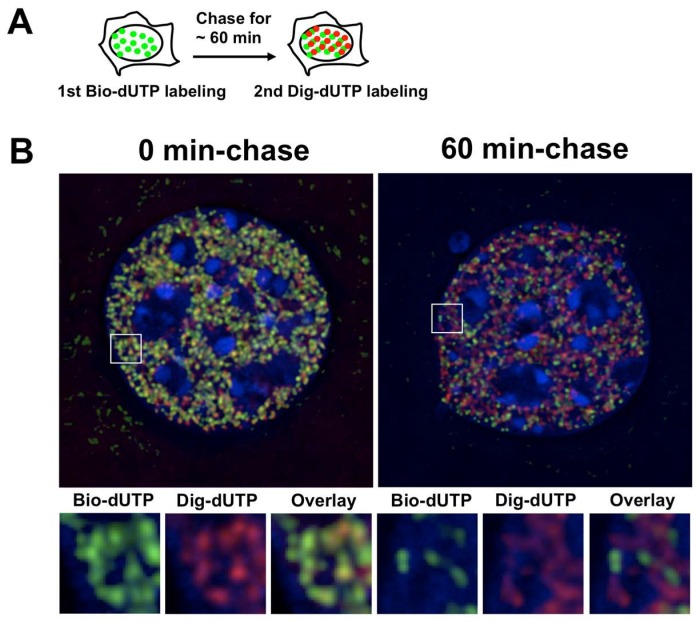
Visualization of DNA replication progression by labeling with digoxigenin-dUTP and biotin-dUTP. (**A**) Cells synchronized at the G1/S border were labeled with both nucleotide analogues simultaneously (0 min chase, **left**). Cells at the G1/S border are first labeled with digoxigenin-dUTP, cultured for 60 min, and labeled with biotin-dUTP (**right**). Incorporated nucleotide analogues are detected with anti-digoxygenin-conjugated rhodamine (**red**) and avidin-FITC (**green**) [18]. Alternatively, cells can be labeled with IdU/CldU and subjected to immunofluorescent detection to visualize the progression of DNA replication [3]; (**B**) Complete separation of digoxygenin- and biotin-dUTP labeled chromosomal regions occurs after 60 min of chase, resulting in no yellow signals in the merged image.

Nucleoside analogues such as CldU and IdU are incorporated into newly synthesized DNA and visualized as replication foci in the nucleus under the conventional light microscope. Pulse-chase-pulse replication foci experiments (5 min–labeling with CldU followed by 5 min–labeling with IdU) have shown that spatial separation of differentially labeled foci in the nucleus requires an approximately 60 min chase period that is species independent [44,45,46,47]. Several hundred foci are generally found per nucleus, almost all of which follow this “60 min rule” regardless of when they appear in S phase [45] (Figure 3A,B). Based on these observations, it has been proposed that the time to complete replication of individual replication units (possibly replicon clusters) is 60 min and activation of neighboring units occurs sequentially every 60 min as S phase progresses. If that is the case, several CTRs with different replication timing should form a stair-shaped domain. However, in reality, replication domain structures are generally divided into two types of CTRs; early and late CTRs.

What is the cause of this discrepancy? It is possible that the 60 min interval only reflects the time required to resolve newly replicated regions in the nucleus at the level of conventional light microscopy, and does not reflect activation of neighboring replication units in most cases. Recent studies using super-resolution light microscopy provided us a totally different view of replication foci that are greater in number and smaller in size. Although super-resolution light microscopy has not yet been applied to pulse-chase-pulse experiments, it is likely that the 60 min rule will be revised by the application of this new technology [48,49,50,51]. Replication domain data from microarrays and NGS technologies are computationally smoothed over a several hundred Kb-window, which may potentially mask the structural complexity of the raw data. This possibility seems unlikely, however, considering that the smoothing window size (typically ~300 Kb) is well below of the estimated size of a single replication focus (~1 Mb).

DNA fiber experiments provide some clues to resolve this discrepancy. Clustered initiation sites spaced at ~150 Kb are often observed at chromosomal regions replicated at the onset of S phase [3]. In these regions, large chromosomal segments are replicated in a relatively short period of time as discussed above (e.g., five evenly spaced origins can replicate nearly 1 Mb–sized chromosomal segment within 1 h if replication forks progress bidirectionally at the speed of 1.5 Kb/min), which may account for the formation of large-sized CTRs. On the other hand, researchers failed to detect obvious clustering of initiation sites in regions adjacent to the primary activated clusters, with some exceptions [52]. Replication forks from the origins at the edge of the primary cluster keep extending without new origin activation in nearly half of the DNA fiber molecules tested [52]. There are indeed some initiation sites activated later on both sides of the primary clusters, but those generally do not seem to be clustered [52,53]. Taken together, it is speculated that early CTRs, whatever their size, almost always terminate replication within the first 1–2 h of S phase and forks at the edge of the CTRs keep extending thereafter to fill the gap between subdomains or to form TTRs.

## 7. Close Relationship between Replication Domains and Three-Dimensional (3D) Genome Organization

Chromatin conformation capture methods such as Hi-C quantify long-range chromatin interactions and are used to analyze the 3D chromatin organization not only at the level of local interactions between promoters and enhancers but also at the level of higher-order chromatin folding [54]. Principal component analysis of Hi-C data divides the genome into two types of compartments, called A and B, which can be further divided into topologically associating domains (TADs) [14]. The A compartments are generally found to be associated with transcriptionally permissive euchromatin, and the B compartments with heterochromatin. Very interestingly, the A and B compartments correlate well with early and late replication domains, respectively [15,16]. When replication domain reorganization occurs in response to differentiation stimuli, a corresponding A/B compartment switch might also occur [42]. Preferential interactions within compartments (A with A, and B with B) seen in Hi-C data indicate that functionally different chromosomal domains occupy distinct spaces within the nucleus, which is consistent with the microscopic observation that early and late replication foci are segregated into distinct nuclear compartments.

Cell cycle dependent establishment of chromatin interactions coincides with the establishment of replication timing at the TDP [41,55,56], suggesting a mechanistic link in the formation of replication domains and the 3D genome structure. Rap1 interacting factor 1 (Rif1) protein is enriched in late replication domains and removal of this protein leads to perturbation of replication domain structure genome-wide [57,58,59]. Not only normally late replicating domains undergo switching to early replication, but even Rif1-unbound early replicating domains undergo switching to late replication. Moreover, chromatin interaction patterns (both within and between replication domains) established during early G1 are also perturbed by Rif1 deletion [59]. Taken together, this suggests that Rif1 might assist in linking domain-wide regulation of replication timing and the 3D genome organization.

An important but unanswered question is whether replication domain reorganization precedes or follows A/B compartment switching during cell differentiation. Analysis of replication domain organization and chromatin interactions at multiple intermediate differentiation stages would provide a definitive answer as to which is the upstream event.

## 8. A New Step toward Understanding the Biological Significance of Replication Domain Regulation

Existing methodologies to analyze replication domain structure provide either a single-cell resolution view at a handful of chromosomal regions or a genome-wide average view of thousands of cells. The extent of cell-to-cell variability in replication domain organization is thus largely unknown. As different types of chromatin are assembled in different stages of S phase [60], fluctuation in replication domain structure would have significant impact on chromatin structure, thereby affecting gene expression [61]. At the level of replication foci, regions labeled in early S phase in a given cell are labeled again in the following early S phase of the same cells [3], demonstrating the cell-to-cell consistency of replication domain organization. On the other hand, we empirically know that the FISH-based replication-timing assay detects a certain degree of variation in replication timing among cells. For example, in the mouse *Igf2* imprinted region, coordination of asynchronous replication (the paternal homologue replicates earlier than the maternal one) generally occurs over several hundred Kb. However, in a small population (~10%) of cells, this coordination is not observed [62]. This may reflect some technical limitation of the method, but the possibility that replication domain organization varies among individual cells cannot be excluded. To examine whether cell-to-cell variation in replication domain structure exists within a cell population, and to what extent variation exists in the whole genome, it is necessary to develop novel quantitative methodologies enabling genome-wide mapping of replication domains in single mammalian cells. The approach that couples sorting of early and late S phase cells with BrdU-immunoprecipiration cannot be applied to single cell analysis. Alternatively, detecting copy number differences that arise between replicated and unreplicated DNA within a single cell might be a promising approach [10,63,64]. Conventional cell population-based assays generally require 200,000 cells (with 25%–30% of S phase cells) for effective BrdU-IP and it is sometimes difficult to obtain enough cells. Therefore, single cell technologies would not only uncover biologically relevant phenomena hidden in bulk measurements, but also broaden the applications of replication domain analysis. For example, it would enable replication domain analysis of cells in very early embryogenesis that have no in vitro culture model. Furthermore, application of recently developed simultaneous profiling of DNA and RNA method to single cell replication domain analysis will directly address the extent to which gene expression heterogeneity can be explained by cell-to-cell variability in replication domain structure [65].

## 9. Concluding Remarks

It is increasingly recognized that during ontogenesis, developmental gene expression programs are often established on the basis of Mb-sized, multi-genic chromosome units [66,67]. Recent advances in genome-wide technologies have enabled description of such units of chromosomes as A/B compartments and lamin-associated domains (LADs) [14,68]. Because of their close relationship to replication domains [15,16,41], a better understanding of replication domains will lead to a better understanding of other types of domains, and vice versa.
